# All‐In‐One Flexible MXene/PET Films via Scalable Scanning Centrifugal Casting for High Transparency and Ultra‐Wide Multispectral Electromagnetic Responses

**DOI:** 10.1002/advs.202501540

**Published:** 2025-05-14

**Authors:** Tian‐Yu Zhang, Qiang‐Qiang Zhao, Bo Sun, Ibrar Ahmed, Ruijia Liu, Chi Liu, Han Wang, Songfeng Pei, Chang Liu, You Zeng

**Affiliations:** ^1^ Shenyang National Laboratory for Materials Science Institute of Metal Research Chinese Academy of Sciences Shenyang 110016 China; ^2^ School of Materials Science and Engineering University of Science and Technology of China Shenyang 110016 China; ^3^ School of Physical Science and Technology ShanghaiTech University Shanghai 200031 China; ^4^ School of System Design and Intelligent Manufacturing Southern University of Science and Technology Shenzhen 518055 China

**Keywords:** electromagnetic shielding, multifunctionality, MXene‐based film, optical transparency, scanning centrifugal casting

## Abstract

Highly integrated multifunctional materials are crucial for the development of next‐generation aircraft and electronic communication devices. In this study, a novel ultra‐thin MXene film with high optical transparency and broad multispectral electromagnetic responses is first fabricated using the scanning centrifugal casting (SCC) technique. The MXene‐coated PET films significantly integrate transparency, UV‐adsorption, infrared (IR) stealth, and electromagnetic shielding from gigahertz (GHz) to terahertz (THz) frequencies together to address the diverse demands of multifunctional applications. The all‐in‐one integrated flexible films exhibit excellent optoelectronic properties (sheet resistance of 163 Ω sq^−1^ at 82% transparency with a figure of merit of 10.88), outstanding IR stealth (IR emissivity below 55%), and ultra‐broad electromagnetic shielding performance (shielding effectiveness of over 10 dB across GHz to THz frequencies). This remarkable performance is attributed to the intrinsic multifunctionality of MXene, ultra‐thin thickness, horizontal alignment of nanosheets, and strong interfacial interactions achieved during the SCC process. These all‐in‐one flexible MXene films exhibit great potential for applications in aircraft windows, wearable electronics, and next‐generation communication technology.

## Graphical abstract

In this study, a novel all‐in‐one MXene/PET film with a highly aligned and compact structure is fabricated via a scanning centrifugal casting method. The resulting film exhibits high optical transparency, low sheet resistance, excellent infrared stealth, and ultra‐broadband electromagnetic shielding across GHz to THz frequencies, highlighting its great potential for next‐generation aerospace windows and advanced communication technologies.

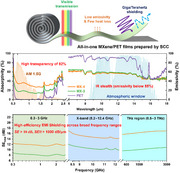

## 1 Introduction

With the rapid advancements in automatic drive, virtual/augmented reality (VR/AR), and artificial intelligence (AI) technologies, sixth‐generation (6G) wireless communication has garnered significant attention due to its fast internet speeds, low latency, and broad application scenarios spanning space, air, ground, and sea systems.^[^ ^1^ ^]^ Unlike 4G/5G which operates at frequency bands of 0.45–6 GHz or 26–40 GHz, 6G communication utilizes higher signal frequency bands reaching up to 0.1–10 THz.^[^ ^2^ ^]^ This shift makes it necessary to develop high‐efficiency electromagnetic interference (EMI) shielding or protection materials with ultra‐broad frequency responses for meeting gradually‐increasing demands of various fields.^[^ ^3^ ^]^ Especially, in some critical applications such as aircraft windows, medical diagnostic equipment, and security inspection apparatuses, the high optical transparency of materials is also indispensable for clear visual observation. For instance, aircraft windows are evolving from traditional metal oxide‐coated glass or embedded metal grids toward integrated multifunctional systems designed to meet the growing demands of next‐generation aerospace platforms, including lightweight construction, efficient ultraviolet shielding, and reduced observability through infrared stealth or thermal camouflage.^[^ ^4^ ^]^ Highly‐integrated multifunctionalities with optical transparency and ultra‐broad electromagnetic responses have become an inevitable trend for advanced materials.^[^ ^5^ ^]^

Transparent and electrically conductive films have undergone rapid development over the past decade, driven by the growing demand for flexible and foldable touchscreens and mobile panels.^[^ ^6^ ^]^ Various conductive fillers, including metal nanoparticles,^[^ ^7^ ^]^ metallic oxides (e.g., indium tin oxide, ITO),^[^ ^8^ ^]^ carbon nanotubes (CNTs),^[^ ^9^ ^]^ silver nanowires or grids,^[^ ^10^ ^]^ graphene,^[^ ^11^ ^]^ and conductive polymers,^[^ ^12^ ^]^ have been deposited, assembled, or transferred onto flexible transparent substrates such as polyethylene terephthalate (PET) or polyimide (PI), consequently exhibiting high surface electrical conductivity and high transparency. These conductive films are also suitable for electromagnetic interference (EMI) shielding applications due to their high conductivity and resulting strong electromagnetic wave reflection. For instance, ITO‐coated films achieve an EMI shielding effectiveness (EMI SE) of 27 dB in the X‐band (8–12 GHz) with 80% transparency.^[^ ^13^ ^]^ Similarly, single‐walled carbon nanotube (SWCNT) films exhibit an EMI SE of 33 dB at 80% transparency,^[^ ^14^ ^]^ monolayer graphene films achieve 2.3 dB at 97% transparency,^[^ ^15^ ^]^ and polyaniline films demonstrate 5.8 dB at 58% transparency.^[^ ^16^ ^]^ However, these studies primarily focus on visible‐light transparency at 550 nm, electrical conductivity, and EMI shielding performance in narrow frequency ranges.^[^ ^17^ ^]^ The multispectral and broad electromagnetic responses of these conductive films have rarely been investigated, owing to the simple chemical composition and inherent single‐frequency response of these conductive fillers.^[^ ^18^ ^]^

Theoretically, optical transparency and electromagnetic (EM) responses of materials depend on their interactions with incident waves. The wavelengths for visible light, gigahertz (GHz), and terahertz (THz) bands lie in 380–700 nm, 1–1000 mm, and 30–3000 µm, respectively, requiring that materials possess matched responses from bond vibration, carrier concentration, and plasmon resonance.^[^ ^19^ ^]^ In that case, highly‐integrated compositions and microstructures are indispensable for achieving high transparency and broad EM responses. Fortunately, MXene (M_n+1_X_n_T_x_), a family of 2D transition metal carbides, nitrides, or carbonitrides,^[^ ^20^ ^]^ exhibit tunable chemical composition, complex bonding, functional groups, layered structures, high electrical conductivity, adjustable layer spacing, and high density of states at the Fermi level,^[^ ^21^ ^]^ enabling the unprecedented integration of multifunctionalities and EM wave interactions across ultraviolet (UV),^[^ ^22^ ^]^ visible light (Vis),^[^ ^23^ ^]^ infrared (IR),^[^ ^24^ ^]^ GHz,^[^ ^25^ ^]^ and THz frequency bands.^[^ ^26^ ^]^ For example, transparent MXene films demonstrate high UV absorption along with transparency exceeding 55%,^[^ ^22^, ^23^ ^]^ effective IR stealth resulting from low IR emissivity,^[^ ^27^ ^]^ satisfactory EMI SE of 10 dB at 8.2–12.4 GHz due to their high electron concentration.^[^ ^28^ ^]^ Additionally, they also exhibit EMI SE of 5.6 dB at THz frequency range, owing to short free‐electron relaxation time (≈15 fs) and localized surface plasmon resonance.^[^ ^29^ ^]^ Notably, conventional MXene films are generally prepared through spinning, spraying, or dip‐coating methods,^[^ ^30^ ^]^ which face challenges of coarse surfaces, loose structure, and low production efficiency. Recently, we developed a scanning centrifugal casting (SCC) technique that synergistically combines centrifugal and shear forces to fabricate large‐area, highly aligned graphene films with exceptional uniformity.^[^ ^31^ ^]^ Building upon this advancement, we have extended the SCC technique to the fabrication of MXene films for the first time. This approach offers a scalable and effective pathway for producing high‐quality MXene films that integrate high optical transparency with ultra‐broadband electromagnetic response. To the best of our knowledge, such a combination of aligned structure, multifunctional integration, and scalable processing has not been reported previously.

This work aims to fabricate highly integrated flexible MXene‐based films with high transparency and broad electromagnetic responses. A large‐area Ti_3_C_2_T_x_ MXene‐coated PET film was fabricated using the SCC method. Their microstructures, multispectral properties (UV–vis and IR), electrical conductivity, and electromagnetic responses at GHz and THz bands were investigated in detail. The MXene/PET films exhibit highly integrated multifunctionalities, including selective UV absorption, high optical transparency, satisfactory IR stealth, and high‐efficiency electromagnetic shielding. The remarkable performance is attributed to the intrinsic MXene multifunctionality, ultra‐thin thickness, smooth surfaces, high alignment, compact contact, and strong interfacial interactions.

## 2 Results and Discussion

### 2.1 Preparation and Microstructures of MXene/PET Films

**Figure 1:**
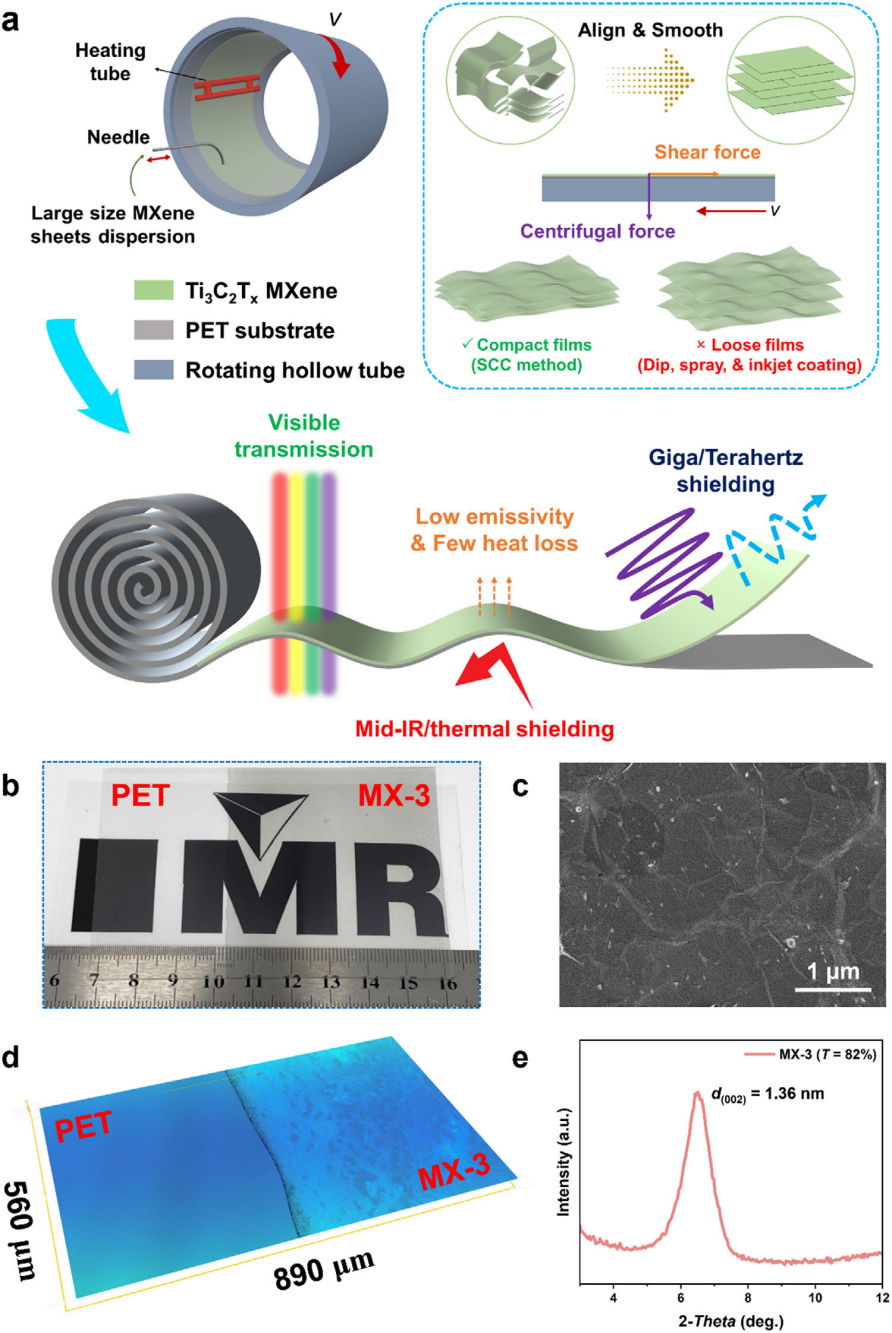
a) Schematic illustration of the preparation process for Ti_3_C_2_T_x_ MXene/PET films using the scanning centrifugal casting (SCC) technique. b) Optical photograph, c) top‐view SEM image, d) 3D surface profile, and e) XRD pattern of the MXene/PET (MX‐3) film.

**Figure** **1** illustrates the preparation schematics and microstructures of Ti_3_C_2_T_x_ MXene/PET films fabricated through scanning centrifugal casting (SCC). The ultrathin and large size MXene nanosheets, with a thickness of ≈2 nm and a lateral size of ≈4 µm (see Figure S1, Supporting Information), were well‐dispersed in aqueous solution during the in‐situ synthesis process due to their high hydrophilic properties.^[^ ^32^ ^]^ The MXene‐containing solution was injected through a needle and deposited onto the inner walls of the rotating tube (see Figure 1a). The deposited MXene nanosheets were subjected to both centrifugal and shear forces: the centrifugal forces compress the MXene nanosheets along the radial direction to form compact and dense films, while the shear forces align the nanosheets along the circular tangential direction to reduce surface roughness and obtain smooth surfaces.^[^ ^31b,c^ ^]^ The compact structures and smooth surfaces are beneficial for enhancing optical transparency, electrical conductivity, and electromagnetic performance.

As shown in Figure 1b, the fabricated MXene/PET (MX‐3) film exhibits satisfactory optical transparency, which is attributed to the ultrathin MXene layer (8.36 nm listed in **Table** **1** of the Experimental Section) and the compact structure formed under the centrifugal forces of the SCC process. The morphological SEM image in Figure 1c reveals that the film possesses a highly flat and smooth surface due to horizontal alignment of the MXene layer induced by shear forces (also see Figure S2, Supporting Information). The flat and smooth surface facilitates reduced surface light scattering, thereby enhancing optical transparency.^[^ ^33^ ^]^ The average surface roughness (*R_a_*) measured using a 3D profilometer is only 2 nm (see Figure 1d and Figure S3, Supporting Information), indicating that the deposited MXene nanosheets were well flattened and aligned under centrifugal and shear forces. The sharp (002) peak at 6.503° and the small interlayer spacing of 1.36 nm shown in Figure 1e also confirm the highly dense and aligned structures of the MXene layer.^[^ ^34^ ^]^ Therefore, the MXene/PET films fabricated via the SCC method exhibit aligned structures, ultrathin thickness, and smooth surfaces, facilitating high optical transparency and superior electromagnetic performance.

**Table 1:** MXene/PET films prepared using SCC method.

Sample	Feeding speed [mm s^−1^]	Deposition time [s]	Thickness of MXene layer^a)^ [nm]	Transparency
MX‐1	16	7.5	2.83	93%
MX‐2	8	15	5.00	89%
MX‐3	4	30	8.36	82%
MX‐4	4	60	17.1	66%
MX‐5	4	90	33.5	45%

^a)^ The thickness of MXene layer was obtained through transparency following Equation S1, Supporting Information.

### 2.2 Optical and Electrical Properties of MXene/PET Films

**Figure 2:**
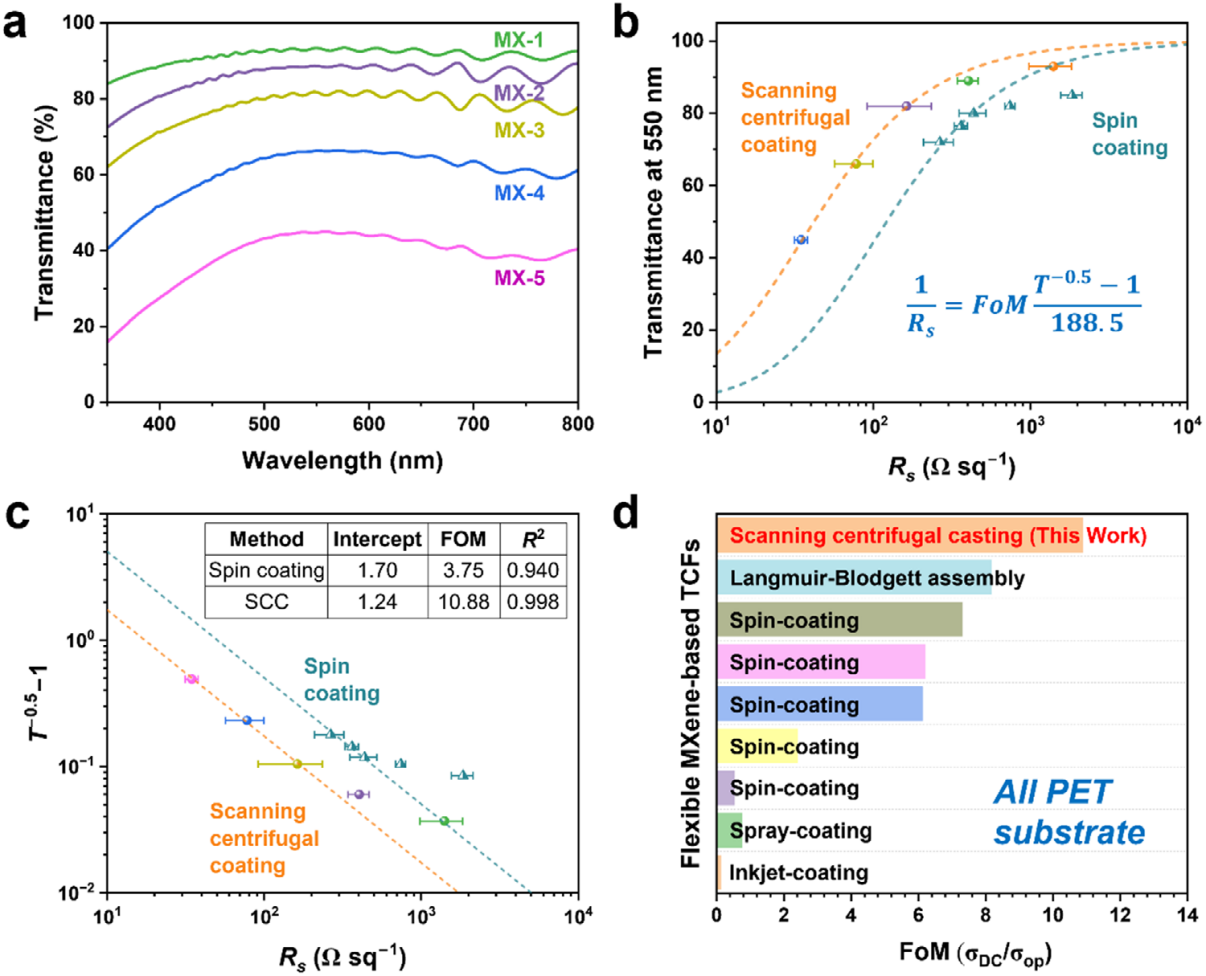
a) UV–vis spectra, b) optical transparency (transmittance at 550 nm) as a function of sheet resistance, and c) corresponding figure of merit (FoM) for the MXene/PET films prepared using the SCC method, and d) FoM comparison between various MXene/PET films.

The optical transparency and electrical properties of the MXene/PET films were measured and shown in **Figure** **2**. As depicted in Figure 2a,b, the transmittance (*T*) gradually decreases with increasing thickness of the MXene layer (from 2.83 nm for MX‐1 to 33.5 nm for MX‐5, as shown in Table 1 of the Experimental Section), accompanied by a decrease in sheet resistance. Balancing high transparency with low sheet resistance is critical for the practical application of transparent conductive films (TCFs). The figure of merit (FoM), defined as the ratio of electrical conductivity (*σ*_DC_) to optical conductivity (*σ*_op_),^[^ ^35^ ^]^ is commonly used to evaluate the performance of TCFs by simultaneously accounting for both transparency and electrical conductivity, as represented in Equation S3, Supporting Information.^[^ ^36^ ^]^ In Figure 2c, the data fit well with Equation S3, Supporting Information, and the calculated FoM value for the SCC‐fabricated MXene/PET films is ≈10.88 (with a coefficient of determination *R* ^2^ = 0.998), higher than the value of 3.75 for MXene/PET films fabricated via the conventional spin‐coating method. This result indicates that the SCC method is highly effective in fabricating high‐quality MXene/PET films with high optical transparency and low sheet resistance.

It can also be seen from Figure 2b that, at the same level of transparency, the films prepared using the SCC method exhibit lower sheet resistance (*R*_s_) compared to those prepared via the spin‐coating method. This superior electrical conductivity is primarily attributed to the highly aligned and compact stacking of MXene nanosheets induced by centrifugal and shear forces during the SCC process.^[^ ^31b^ ^]^ In our work, the MX‐3 and MX‐4 films exhibit sheet resistances of 162.9 and 77.9 Ω sq^−1^ with corresponding transmittance at 550 nm (*T*_550_) of 82% and 66%, respectively (also see Table 1), meeting the requirements for commercial TCF applications.^[^ ^36^ ^]^ Additionally, compared with MXene films prepared using other methods such as dip coating,^[^ ^37^ ^]^ spray coating,^[^ ^38^ ^]^ and inkjet coating,^[^ ^39^ ^]^ as summarized in Table S1, Supporting Information, the SCC‐fabricated MXene/PET films in this work still exhibit a higher FoM value, as shown in Figure 2d. The enhanced transparency and conductivity are primarily attributed to the ultrathin thickness, smooth surface morphology, compact stacking of nanosheets, and reduced contact resistance.

### 2.3 Multispectral Performance of MXene/PET Films

**Figure 3:**
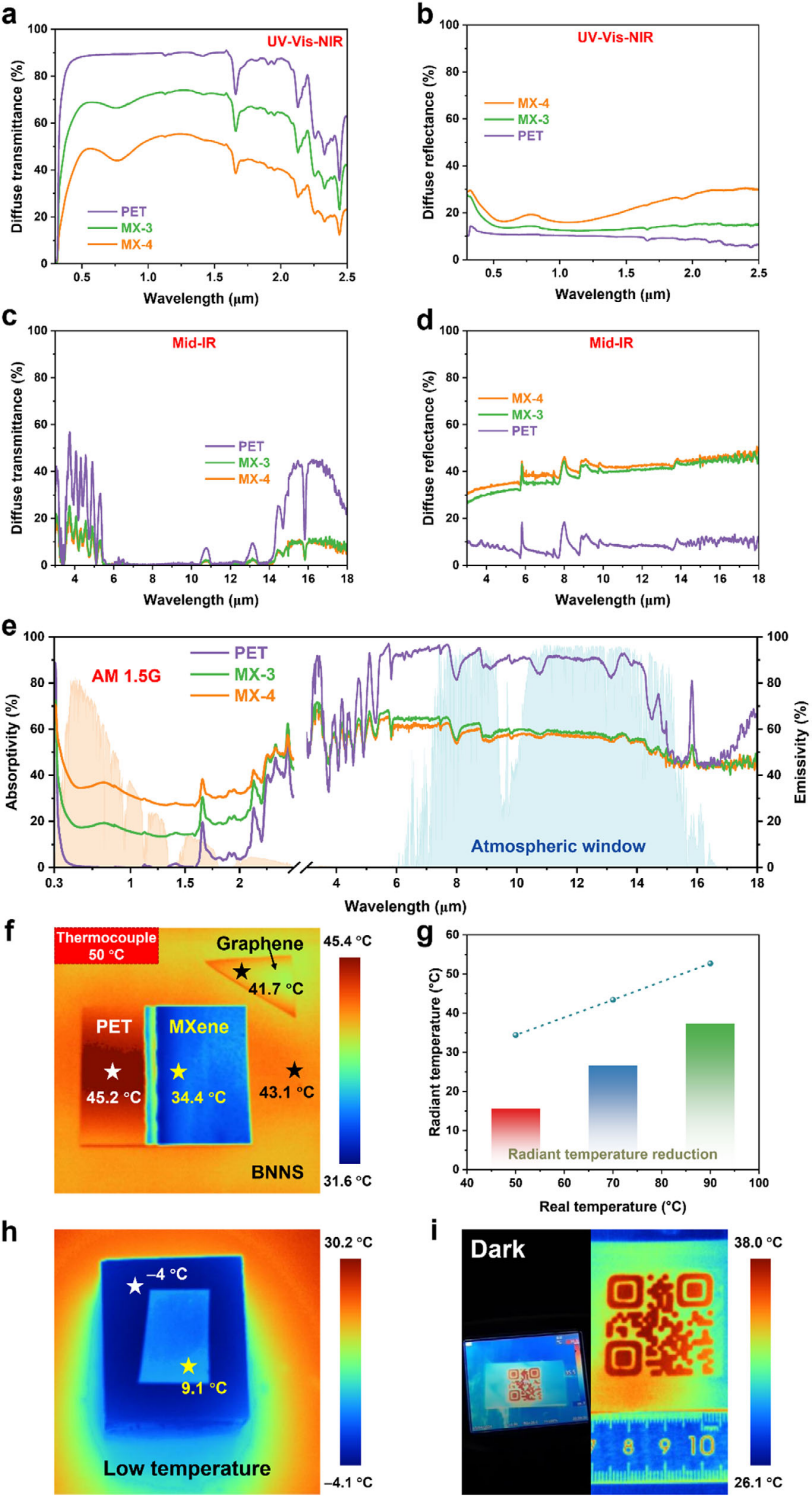
Multispectral performance of MXene/PET films. Diffuse transmittance and reflectance in a,b) UV–vis‐NIR and c,d) Mid‐IR regions. e) Absorptivity and emissivity in a wide spectral range. f) IR thermal images and (g) the corresponding radiation temperatures of MX‐3 film at various surrounding temperatures. IR thermal images of the MX‐3 film paced in h) ice bath and i) dark environment.

The multispectral performance of the MXene/PET films was investigated using UV–vis–NIR and FT‐IR spectrometers equipped with integrating spheres, covering a broad wavelength range from 0.3 to 18 µm and encompassing the ultraviolet (UV), visible, near‐infrared (NIR), and mid‐infrared (MIR) regions (see **Figure** **3**). In the UV region (wavelength: 0.3–0.4 µm) of Figure 3a,b, the MXene/PET films exhibit lower diffuse transmittance and slightly higher diffuse reflectance compared to pristine PET, indicating stronger UV absorption and consistent with our previous findings.^[^ ^23^ ^]^ The strong UV absorption is primarily attributed to the layered structure of MXene, composed of Ti and C layers and surface terminal atoms (F, O, and H), which exhibit a bandgap (0.92–1.75 eV) that matches the energy of UV photons, enabling high‐efficiency absorption.^[^ ^23^, ^40^ ^]^ In contrast, in the visible‐light region (wavelength: 0.4–0.76 µm), the MXene/PET films exhibit enhanced diffuse transmittance due to the mismatched bandgaps (1.63–3.26 eV for visible light).^[^ ^41^ ^]^ This indicates that MXene/PET films achieve high transparency in the visible range while effectively blocking UV radiation, which is closely associated with the distinct interactions of MXene with incident visible and UV light.^[^ ^22^, ^23^ ^]^ In the near‐infrared (NIR) region (wavelength: 0.76–2.5 µm), both PET and MXene/PET films exhibit decreased transmittance and enhanced absorption, primarily due to strong interactions between chemical bonds and incident NIR light.^[^ ^27b^ ^]^ Additionally, across the entire UV–vis‐NIR spectrum, the MX‐3 films show higher transmittance and lower reflectance compared to the MX‐4 films, which is attributed to their thinner MXene layer (8.36 nm) and weaker interactions between MXene and incident photons.

In the mid‐IR region (3–18 µm) of Figure 3c,d, all the films exhibit low transmittance, indicating strong IR absorption due to drastic vibrations of chemical bonds, including C═O and C─O─C stretching, and C─H bending.^[^ ^42^ ^]^ Unlike the matched bandgaps for UV absorption, the vibration of chemical bonds is dominate for the mid‐IR absorption. Additionally, Figure 3d shows the enhanced diffuse reflectance of the MXene/PET films compared to PET, resulting from the high intrinsic electrical conductivity of MXene.^[^ ^27^, ^43^ ^]^ Figure 3e illustrates the absorptivity of the MXene/PET films across the entire multispectral range, revealing their highly selective optical behavior—exhibiting low absorption (i.e., high transparency) specifically in the visible‐light region. Using FT‐IR measurements with integrating spheres, the emissivity of the MXene/PET films was determined and equal to their absorptivity in thermal equilibrium conditions based on Kirchhoff's law.^[^ ^3b^ ^]^ It can be seen that the MXene/PET films exhibit much lower emissivity below 55% compared to pristine PET, indicating that the MXene‐coated films have a reduced thermal radiation capacity and emit less IR radiation even at the same temperatures. This low emissivity is primarily attributed to constrained electron movement, reduced charge fluctuation, and the layered structure of MXene.^[^ ^27a,b^ ^]^

The low emissivity of the MXene/PET films implies the reduced thermal radiation capacity, highlighting their potential applications in IR stealth, camouflage, active heating, and de‐icing. As shown in Figure 3f, the MX‐3 films exhibit the lowest IR thermal imaging temperature of 34.4 °C among all the heated materials (PET, graphene, and BNNS), which is significantly lower than the actual temperature of 50 °C. This remarkable temperature deviation is primarily attributed to the low IR thermal radiation, which affects temperature detection and results in an IR stealth phenomenon.^[^ ^3b^ ^]^ In our work, the temperature deviation of 15.6 °C observed in the MX‐3 films is applicable to thermal camouflage for observation cabins equipped with electronic devices, where an actual surface temperature of 50 °C appears as 34 °C, reducing the thermal signal to near ambient temperature and making it nearly indistinguishable from the surrounding environment. Notably, the IR stealth performance is influenced by multiple factors, including the intrinsic emissivity, surface roughness, oxidation degree, and ambient environmental conditions.^[^ ^44^ ^]^ In many scenarios, IR stealth across a wide temperature range (−60 to 100 °C) is also required.^[^ ^3b^ ^]^ As shown in Figure 3g, the temperature deviation of the MXene/PET films increases proportionally with the ambient temperature in the range of 50 to 90 °C. Similarly, when the MXene/PET film was placed on ice at −4 °C, it exhibited obvious IR stealth with a temperature deviation of 13.1 °C. Even in dark environments, the low‐emissivity MXene/PET films emit less thermal radiation compared to high‐emissivity substrates, enhancing contrast for clear pattern detection (see Figure 3i and Movie S1, Supporting Information). Additionally, the MXene/PET films demonstrate highly‐efficient electrical and photothermal heating as well as de‐icing capabilities due to the low thermal radiation of MXene (see Figure S4, Supporting Information). Consequently, the MXene/PET films exhibit multispectral characteristics, including high UV absorption, low visible‐light absorption (high transparency), and low mid‐IR absorption (IR stealth), which are closely associated with the distinctive interactions between MXene and incident light.

### 2.4 Electromagnetic Response Performance

**Figure 4:**
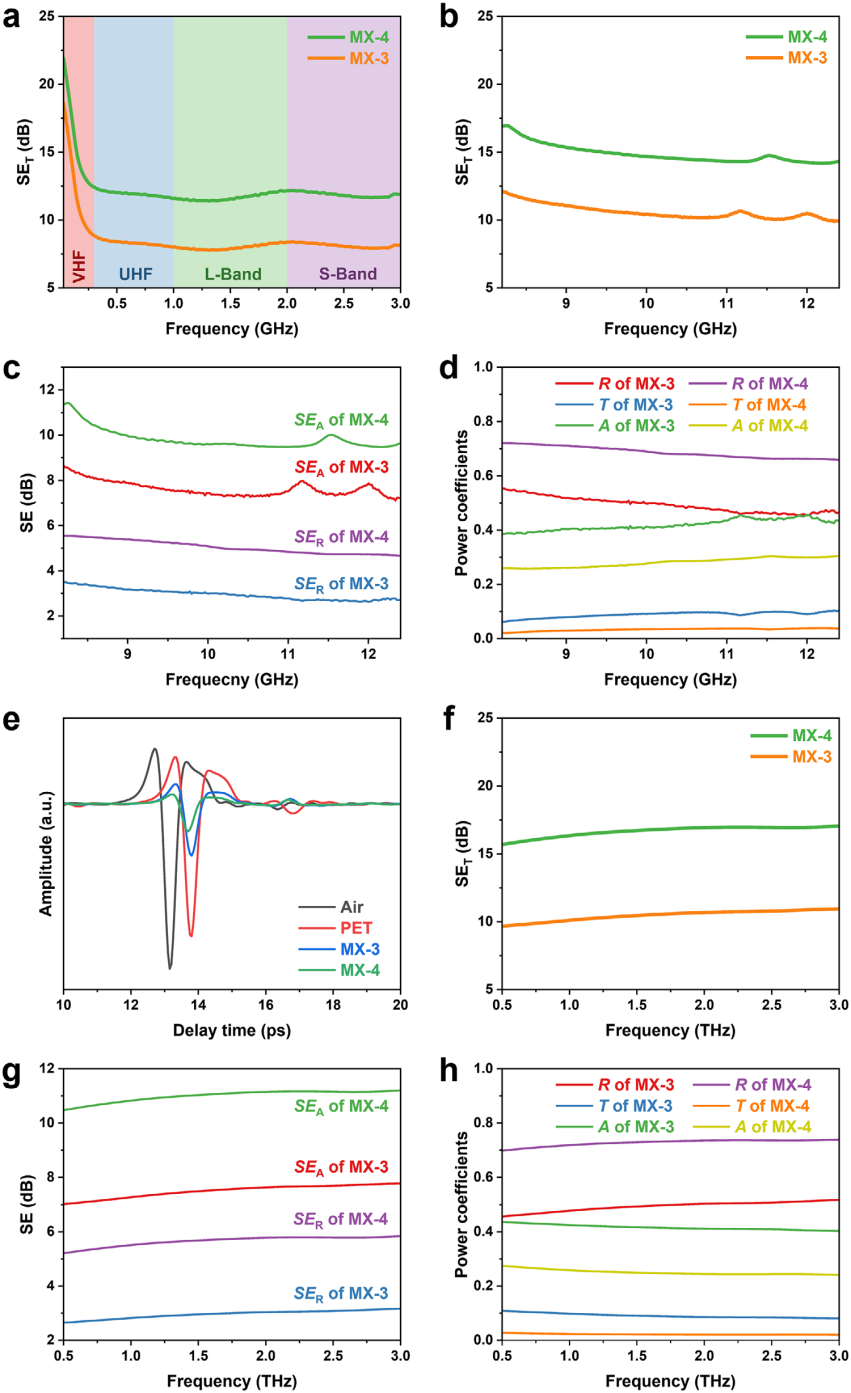
EMI shielding performance of transparent MXene/PET films at a) 0.03–3 GHz, b–d) 8.2–12.4 GHz, and e–h) 0.5–3 THz frequencies. b,c) Shielding effectiveness of total (*SE*_T_), absorption (*SE*_A_), and reflection (*SE*_R_), and d) corresponding power coefficients of reflection (*R*), absorption (*A*), and transmission (*T*) at 8.2–12.4 GHz frequencies. e) Amplitude of THz measurement signals versus delay time. f,g) Shielding effectiveness and h) power coefficients at 0.5–3 THz frequencies.

The electromagnetic response behavior of transparent MXene/PET films (MX‐3 and MX‐4) was investigated across frequency ranges of 0.03–3 GHz, 8.2–12.4 GHz, and 0.5–3 THz, as shown in **Figure** **4**. Theoretically, the electromagnetic response strongly depends on the interaction between the MXene/PET films and the incident electromagnetic waves.^[^ ^45^ ^]^ According to Faraday's law of electromagnetic induction,^[^ ^46^ ^]^ high‐frequency electromagnetic fields in and around the electrically‐conductive MXene films inevitably induce a non‐uniform current distribution across the cross‐section, with current density concentrating near the surface —a phenomenon known as the skin effect.^[^ ^47^ ^]^ The skin effect is generally represented by the skin depth, which is inversely proportional to the square root of frequency and electrical conductivity.^[^ ^47^ ^]^ As the frequency increases, the skin depth decreases, causing more current to concentrate at the surface (i.e., a more pronounced skin effect), which results in stronger reflection of electromagnetic waves.^[^ ^48^ ^]^ As shown in Figure 4a, the transparent MXene/PET films demonstrate EMI shielding performance in the 0.03–3 GHz frequency range, primarily due to the skin effect and enhanced surface current density that promote electromagnetic wave reflection. The MX‐4 films exhibit higher shielding effectiveness (*SE*) of 12 dB compared to 8 dB for the MX‐3 films, which is attributed to more pronounced surface current density and resultant stronger electromagnetic wave reflection.

At higher frequencies (8.2–12.4 GHz, as shown in Figure 4b), the MXene/PET films exhibit higher *SE* compared to the 0.03–3 GHz range, indicating improved shielding performance at elevated frequencies. This enhancement is primarily attributed to more pronounced skin effect and increased electromagnetic wave reflection at higher frequencies. As shown in Figure 4c, the MX‐4 films display higher values of total shielding effectiveness (*SE*_T_), reflection effectiveness (*SE*_R_), and absorption effectiveness (*SE*_A_) than those of the MX‐3 films. These improvements arise from the higher surface current density (contributing to enhanced reflection) and greater MXene thickness (enabling increased absorption and energy attenuation through mechanisms such as multiple reflections,^[^ ^49^ ^]^ cavity resonance,^[^ ^50^ ^]^ dipole polarization, and interfacial polarization^[^ ^45b^ ^]^). To further elucidate the shielding mechanism, the power coefficients of reflection (*R*), absorption (*A*), and transmission (*T*) were calculated and shown in Figure 4d. The MX‐4 films exhibit the *R* value as high as 0.70, indicating that ≈70% of incident waves are reflected and confirming reflection as the dominant shielding mechanism. In contrast, the MX‐3 films show a lower *R* value (0.47) and higher *A* value (0.45), implying that more electromagnetic waves penetrate the film and are attenuated via multiple reflections and dielectric losses.^[^ ^45^, ^51^ ^]^

At higher frequencies (0.5–3 THz), the MXene/PET films still exhibit satisfactory EMI shielding performance, as evidenced by the pronounced reduction in signal amplitude (rapid energy attenuation) shown in Figure 4e. As illustrated in Figure 4f,g, the MX‐4 films show higher *SE*_T_ of 17 dB than the MX‐3 films, with corresponding increases in both *SE*_A_ and *SE*_R_, consistent with that in 8.2–12.4 GHz. Notably, compared with the 8.2–12.4 GHz range, both MX‐4 and MX‐3 films exhibit higher *R* values in THz range (0.72 and 0.51, respectively), which is mainly attributed to the enhanced surface current density at THz frequencies and the stronger electromagnetic wave reflection. As shown in Figure 4h, the *R* value for the MX‐4 films is 0.72, much higher than *A* (0.26), further confirming reflection as the dominant shielding mechanism in the THz range. Similarly, the MX‐3 films still retain high *A* (0.40), indicating notable energy attenuation through electromagnetic wave absorption at THz frequencies.^[^ ^29b^ ^]^ Therefore, the MXene/PET films exhibit satisfactory EMI shielding performance across a broad frequency range (0.03–3 GHz, 8.2–12.4 GHz, and 0.5–3 THz), primarily due to the strong interaction with incident electromagnetic waves, elevated surface current density, and efficient electromagnetic wave reflection.

Furthermore, we compared our EMI shielding performance with that of transparent films reported in literature, as shown in Table S2, Supporting Information. In this work, the EMI SE of MXene/PET films are over 10 dB at 8–12 GHz and 0.5–3 THz bands, comparable with that for other films. Notably, the thickness of our MXene layer (MX‐3) is only 8.3 nm, and the resultant shielding effectiveness unit thickness (*SE*/*t*) is up to over 1000 dB µm^−1^ across wide frequency ranges, much higher than that for other transparent films such as CNTs,^[^ ^52^ ^]^ AgNWs,^[^ ^53^ ^]^ and MXene films fabricated using conventional methods.^[^ ^54^ ^]^ This high‐efficiency EMI shielding performance is attributed to the horizontal alignment of MXene, compact structure, and strong interfacial interactions achieved during the SCC process. In addition, the MXene/PET films also exhibit excellent transparency of 82% with a figure of merit of 10.88, higher than other MXene‐based flexible films reported in other studies.^[^ ^38^, ^39^, ^54^, ^55^ ^]^ Moreover, the MXene/PET films also demonstrate high UV absorption and low IR emissivity (IR stealth). Therefore, the all‐in‐one MXene/PET films exhibit highly‐integrated characteristics of high transparency and broad electromagnetic responses, showing great potential to be utilized in many application scenarios.

## 3 Conclusion

In summary, we successfully developed novel ultra‐thin, transparent MXene films with broadband electromagnetic responses by employing a scanning centrifugal casting (SCC) technique. Through systematic characterization, we demonstrated that the resulting MXene/PET films possess a desirable combination of high optical transparency, low sheet resistance (163 Ω sq^−1^ at 82% transmittance), and a figure of merit as high as 10.88. Additionally, the films exhibit excellent infrared stealth capabilities (emissivity below 55%) and outstanding EMI shielding effectiveness over a wide spectral range spanning from the GHz to THz bands. These superior multifunctionalities is due to the distinctive structural advantages achieved through the SCC process, including horizontal alignment, ultrathin thickness, smooth surface, and strong interfacial interactions. This work highlights the significant potential of SCC‐fabricated MXene films for next‐generation applications in transparent EMI shielding, aerospace windows, wearable electronics, and wireless communication technologies.

## 4 Experimental Section

### Materials

Ti_3_AlC_2_ MAX phase powders (average particle size of 37 µm) prepared using pressure‐less sintering were provided by Foshan Xinxi Technol. Co. Ltd. (China). Lithium fluoride (LiF, CP), hydrochloric acid (HCl, AR), and anhydrous ethanol (C_2_H_5_OH, AR) were purchased from Sinopharm Chemical Reagent Co. Ltd. (China). Deionized water (DIW) was obtained from Merck Millipore‐Q water purification system. PET substrates with 250 µm thickness were purchased from Suzhou Southeast Center Electronic Material Co. Ltd. (China). 2D boron nitride nanosheet (BNNS) film (SPA‐TF60) with a thickness of 30 µm was provided by Guangdong Shengpeng Technology Co. Ltd. (China).

### Synthesis of Ultrathin Ti_3_C_2_T_x_ MXene Nanosheets

Ultrathin Ti_3_C_2_T_x_ MXene nanosheets were synthesized by etching Ti_3_AlC_2_ MAX phase using a mixed solution of HCl and LiF.^[^ ^56^ ^]^ First, 3.2 g of LiF was added to 40 mL of HCl aqueous solution with a 9.25% concentration and stirred in a Teflon vessel for 30 min. Next, 2.0 g of Ti_3_AlC_2_ MAX phase powders with a typical layered structure (see Figure S1a, Supporting Information) was slowly added and etched at 40 °C for 24 h. The resultant product was repeatedly washed with DIW to remove residuals by centrifugation at 3500 rpm for 5 min until the pH of the supernatant stabilized at 6. The product was then filtered using cellulose filter membranes with pore size of 0.22 µm. Subsequently, the sediments were dispersed in 100 mL of DIW and ultrasonicated for 15 min to mechanically exfoliate the multilayer‐stacked structures into nanosheets under inert atmosphere in ice bath. After centrifugation at 3000 rpm for 15 min, the supernatant was collected, and a colloidal solution containing ultrathin Ti_3_C_2_T_x_ MXene nanosheets (≈5 mg mL^−1^) was obtained. The prepared Ti_3_C_2_T_x_ MXene nanosheets exhibited characteristic hexagonal crystal structures,^[^ ^23^, ^57^ ^]^ large average lateral size of ≈4 µm, and ultra‐thin thickness of less than 2 nm (see Figure S1, Supporting Information), beneficial to achieving high transparency and efficient electron transport.

### Fabrication of MXene‐Coated PET Films

Flexible and transparent MXene/PET films were prepared using the scanning centrifugal casting (SCC) technique. First, PET films were cleaned and treated in a plasma treatment system (Zhongshan PLS Plasma Technology Co. Ltd. of China) to enhance their hydrophilicity. Next, the treated PET film was fixed onto the inner wall of a rotating hollow tube (RHT) and heated to 80 °C. While the RHT rotated at 1000 rpm, the MXene‐containing solution (5 mg mL^−1^) was injected through a needle and deposited onto the PET substrate. The needle moved back and forth along the rotational axis at speeds of 4–16 mm s^−1^ following a preset control procedure. During deposition, The MXene nanosheets were subject to the centrifugal forces for compact contacts with PET and the shearing forces for aligned structure and smooth surface.^[^ ^31b^ ^]^ After drying, a thin flexible Ti_3_C_2_T_x_ MXene‐coated PET film was obtained. The thickness of MXene layer was well controlled by adjusting the needle feeding speed and deposition time. Consequently, five samples (MX‐1, MX‐2, MX‐3, MX‐4, and MX‐5) were fabricated and listed in Table 1. Additionally, MXene solution with the same concentration was spin‐coated onto PET substrates for comparison.

### Materials Characterization

The morphology and microstructures of the MXene nanosheets and MXene/PET films were characterized using scanning electron microscopy (SEM, Thermo Scientific Verios G4 UC, USA), high‐resolution transmission electron microscopy (HRTEM, FEI Tecnai F20, USA), atomic force microscopy (AFM, Bruker MultiMode 8, Germany), and X‐ray diffractometer (XRD, PANalytical) with Cu Kα radiation (λ = 1.5418 Å) operated at 40 kV. The thickness of MXene layers was evaluated using a UV–vis transmittance method (see Note S1, Supporting Information), due to the limitations of SEM‐based measurements for ultra‐thin films. The surface topography and roughness of the MXene/PET films were evaluated using a 3D profilometer (MFT‐5000, Rtec Instruments, USA).

### Electrical and Multispectral Measurements

The sheet resistance (*R*_s_) and electrical conductivity (*σ*) of the MXene/PET films were measured using a standard four‐probe tester (RTS‐9, 4 Probes Tech Ltd., China). The light absorption and transmission properties of the MXene/PET films over a wide wavelength range (0.3–20 µm) were evaluated using UV–vis–NIR spectrophotometer (JASCO V‐770, Japan) and Fourier transform infrared (FT‐IR) spectrometer (Spotlight 200i, PerkinElmer, USA) equipped with integrating spheres. The transmittance (*T*) at 550 nm was used as the evaluation index for optical transparency. Combined with the corresponding surface electrical resistance (*R*_s_), a figure of merit (FoM) was calculated based on Equations S2 and S3, Supporting Information to assess the optoelectronic properties of transparent and conductive films. In addition, the IR emissivity (*ε*) of MXene/PET films was calculated following the Kirchhoff's law to evaluate their IR stealth performance. Furthermore, IR images and surface radiation temperatures of the specimens placed on plates with various temperatures were recorded using a thermal imager (Fluke Ti32, USA).

### Electromagnetic Interference Shielding Performance Measurements

The EMI shielding performance of MXene/PET films was measured across frequency bands of 30 MHz–3 GHz and 8.2–12.4 GHz (X band) using a vector network analyzer (VNA, Keysight N5242B PNA‐X, USA) equipped with coaxial transmission line and rectangular waveguide holders, following ASTM D4935 and D5568, respectively. The scattering parameters of *S*_11_ and *S*_21_ were recorded, and the coefficients of reflection (*R*), absorption (*A*), and transmission (*T*), along with EMI shielding effectiveness (SE), were calculated based on Equations S4–S9, Supporting Information. For shielding measurements in the THz frequency band, the MXene/PET film was mounted on a circular sample holder with a hollow center (pore diameter: 1.2 cm) and measured using a THz time‐domain spectrometer system (TPS Spectra 3000, TeraView Ltd., United Kingdom). The EMI SE in the THz range was further calculated based on Equations S7–S9, Supporting Information as well.

## Conflict of Interest

The authors declare no conflict of interest.

## Author Contributions

T.‐Y.Z. and Q.‐Q.Z. contributed equally to this work. T.‐Y.Z.: Writing – original draft, Writing – review and editing, Visualization, Methodology, Investigation, Formal analysis. Q.‐Q.Z.: Methodology, Investigation, Formal analysis. B.S.: Formal analysis, Visualization. I.A.: Formal analysis, Visualization. R.L.: Formal analysis, Visualization. C.L.: Resources, Investigation. H.W.: Writing – review and editing, Funding acquisition. S.P.: Resources, Investigation, Supervision. C.L.: Supervision, Funding acquisition. Y.Z.: Writing – review and editing, Supervision, Project administration, Funding acquisition, Conceptualization.

## Supporting information



Supporting Information

Supplemental Movie 1

## Data Availability

The data that support the findings of this study are available from the corresponding author upon reasonable request.
