# Supplementary material for: All‐In‐One Flexible MXene/PET Films via Scalable Scanning Centrifugal Casting for High Transparency and Ultra‐Wide Multispectral Electromagnetic Responses
Source: Adv Sci (Weinh). 2025 May 14;12(27):2501540. doi: 10.1002/advs.202501540 (PMC12279239; doi:10.1002/advs.202501540)
Supplement: Supplementary file 1 — Supporting Information [file ADVS-12-2501540-s002.docx]

**Supporting Information**

**All-in-one Flexible MXene/PET Films via Scalable Scanning Centrifugal Casting for High Transparency and Ultra-Wide Multispectral Electromagnetic Responses**

*Tian-Yu Zhang, Qiang-Qiang Zhao, Bo Sun, Ibrar Ahmed, Ruijia Liu, Chi Liu, Han Wang*, Songfeng, Pei*, Chang Liu, and You Zeng**

T.-Y. Zhang, Q.-Q. Zhao, B. Sun, I. Ahmed, Prof. C. Liu, Dr. H. Wang, Prof. S. Pei, Prof. C. Liu, Prof. Y. Zeng

Shenyang National Laboratory for Materials Science, Institute of Metal Research, Chinese Academy of Sciences, Shenyang 110016, China

E-mails: hanwang@imr.ac.cn; sfpei@imr.ac.cn; yzeng@imr.ac.cn

T.-Y. Zhang, I. Ahmed, Prof. C. Liu, Dr. H. Wang, Prof. S. Pei, Prof. C. Liu, Prof. Y. Zeng

School of Materials Science and Engineering, University of Science and Technology of China, Shenyang 110016, China

Q.-Q. Zhao

School of Physical Science and Technology, ShanghaiTech University, Shanghai 200031, China

Dr. R. Liu

School of System Design and Intelligent Manufacturing, Southern University of Science and Technology, Shenzhen 518055, China

**SUPPLEMENTARY FIGURES**


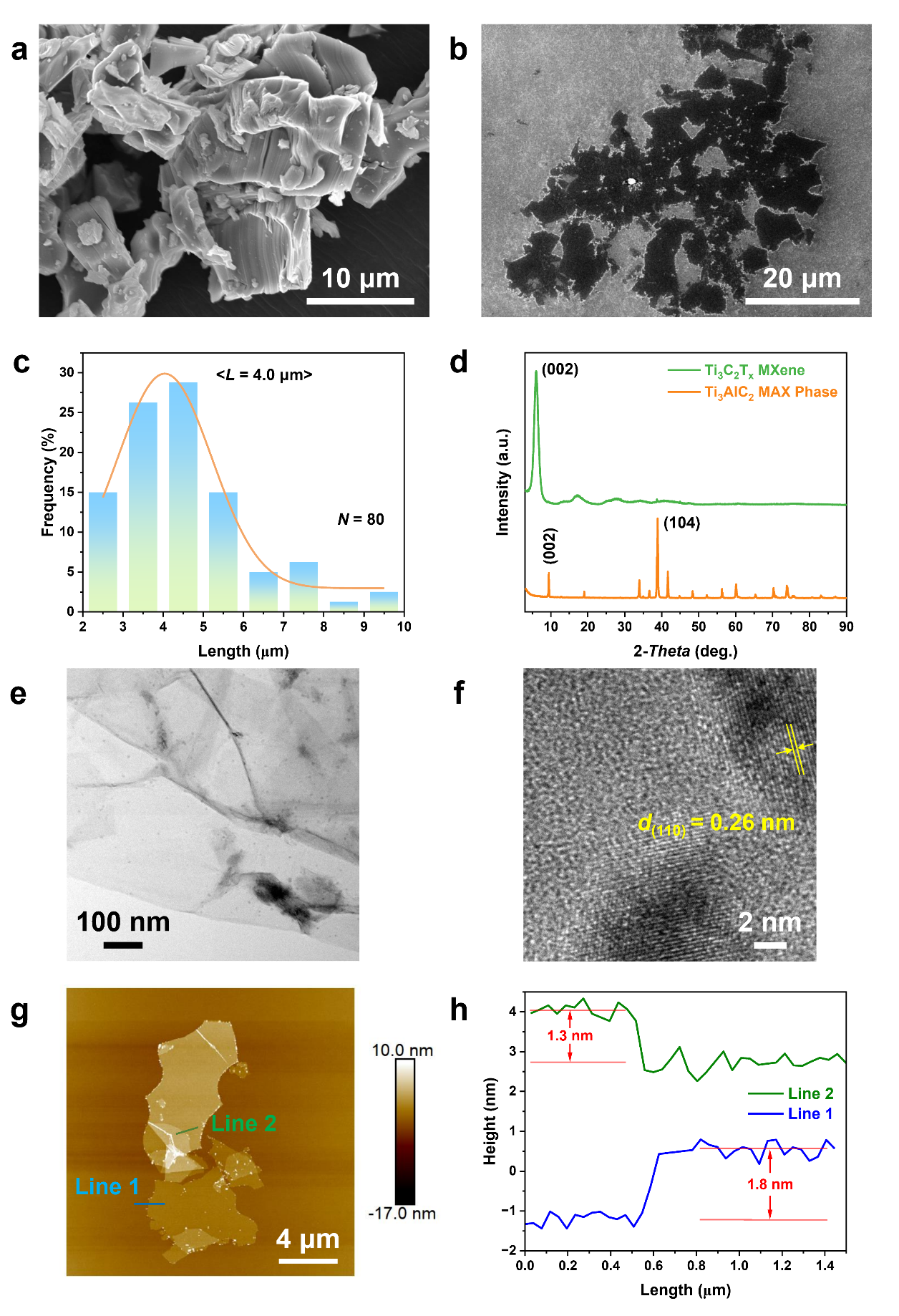


**Figure S1.** Morphology and microstructures of Ti_3_AlC_2_ MAX phase and ultrathin Ti_3_C_2_T_x_ MXene nanosheets. SEM images of (a) Ti_3_AlC_2_ MAX phase and (b) Ti_3_C_2_T_x_ MXene nanosheets. (c) Histogram of MXene flake sizes. (d) XRD patterns, (e) TEM, (f) HRTEM, and (g) AFM images of Ti_3_C_2_T_x_ MXene flakes with (h) thickness of less than 2 nm.


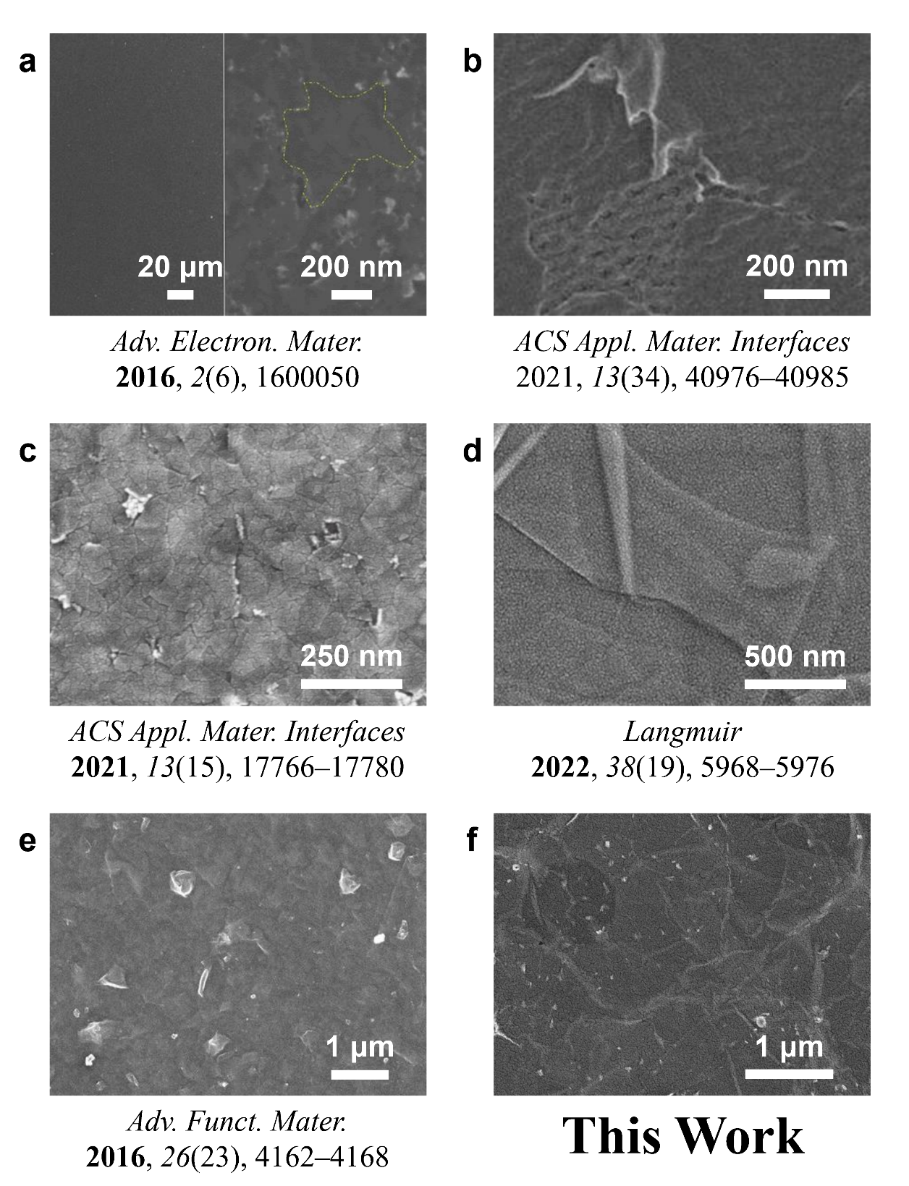


**Figure S2.** SEM comparison of previously published work with our transparent MXene/PET film prepared through scanning centrifugal casting (SCC) method.


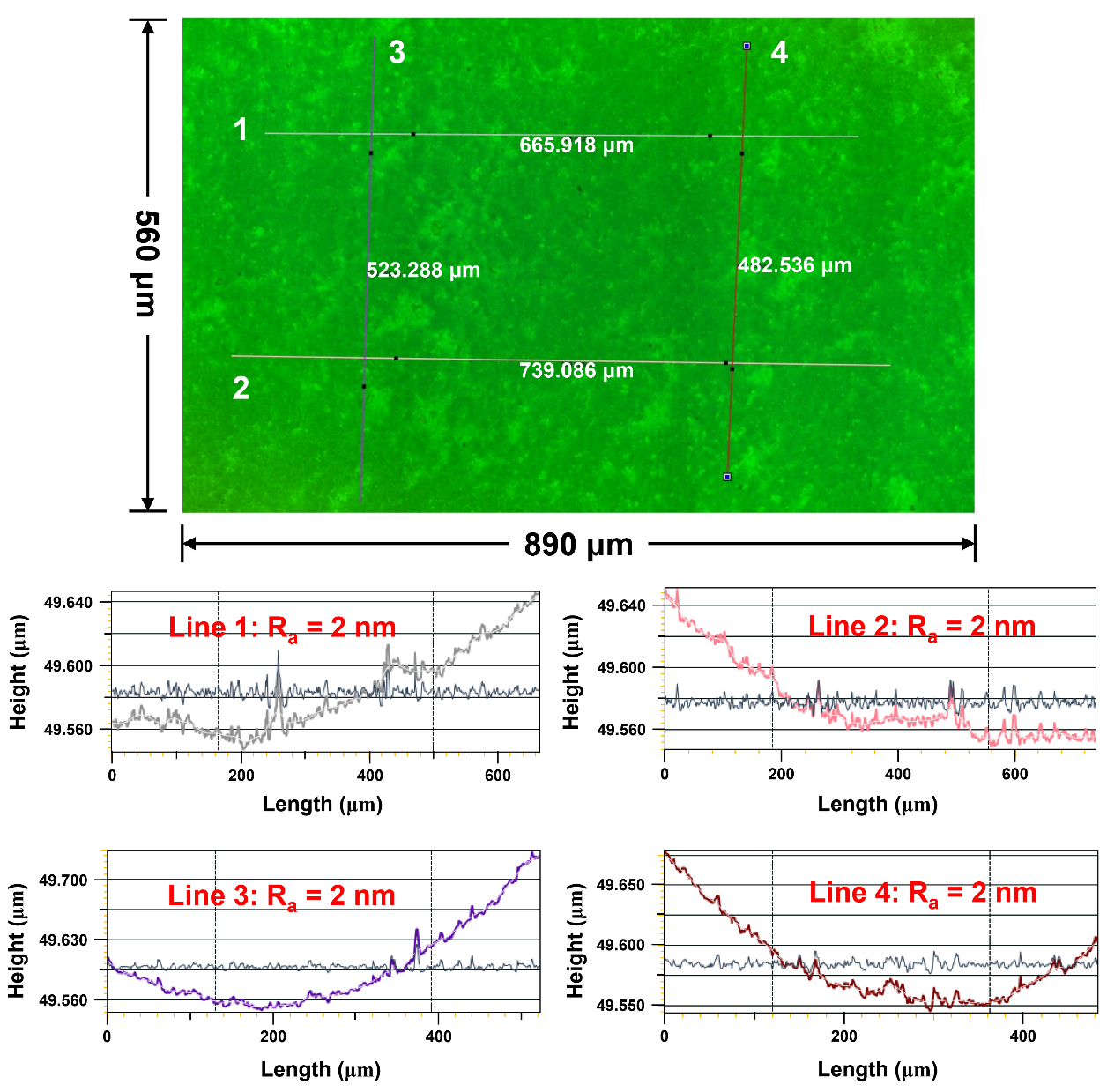


**Figure S3.** 2D surface profiles of the MX-3 film with *T*_550_ = 82%, showing a roughness average (*R_a_*) value of 2 nm.


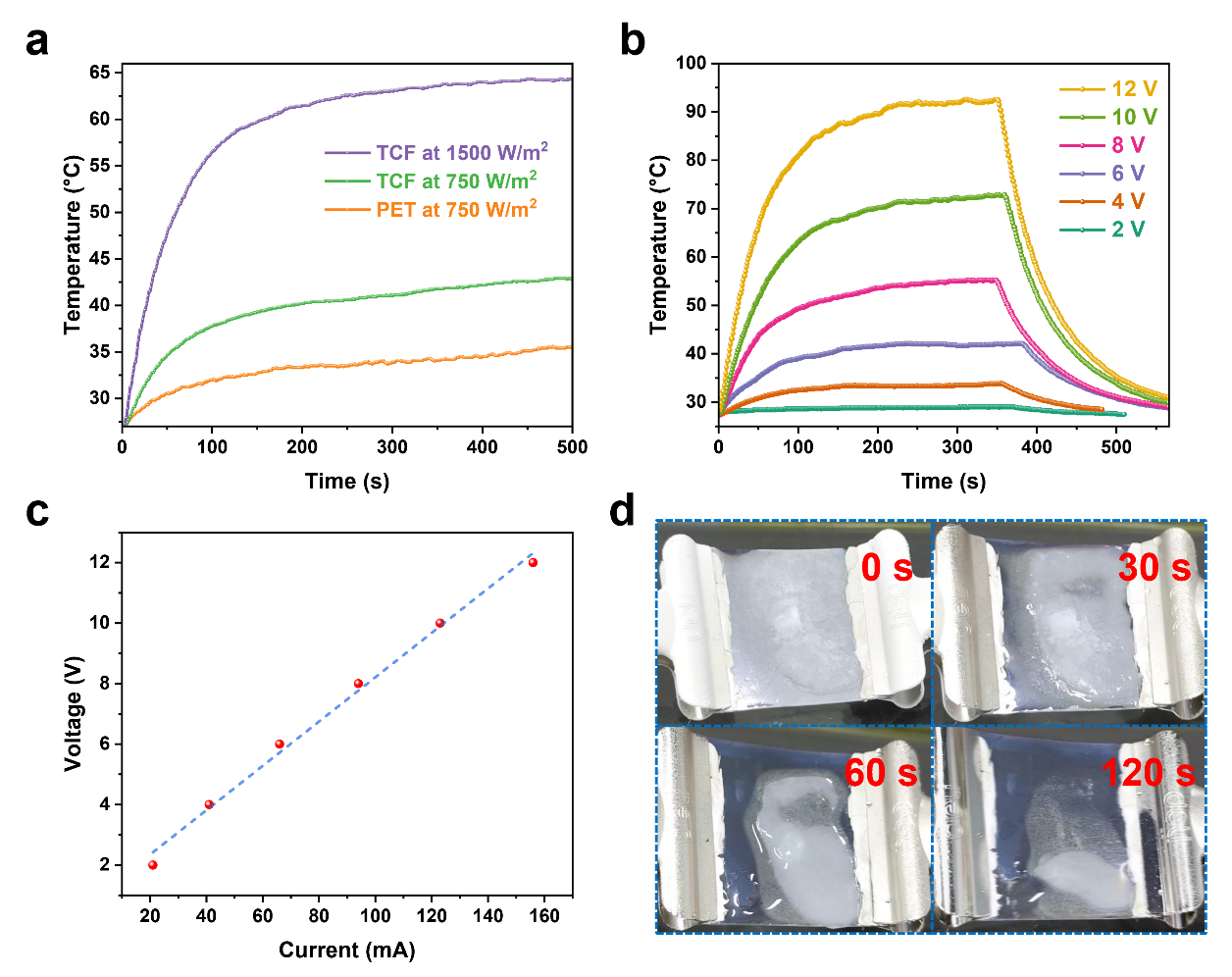


**Figure S4.** Photo-/electro-thermal performance and de-icing applications of MX-4 films. The photo-thermal (a) and electro-thermal (b) curves at various light power densities and voltages, respectively. (c) The current response at different onset voltage, and (d) the electro-thermal deicing process of MX-4 film.

**SUPPLEMENTARY TABLES**

**Table S1.** Comparison of the FoM values for flexible MXene-based transparent conductive films (TCFs) prepared using various methods.

| **Materials** | **Sizes** | **Methods** | ***T*_550_** | **R_s_ (Ω sq^−1^)** | **FoM** | **Ref.** |
| --- | --- | --- | --- | --- | --- | --- |
| Ti_3_C_2_T_x_ | 100~300 nm | Inkjet-printing | 24% | 1500 | 0.12 | [1] |
| Ti_3_C_2_T_x_ | ~500 nm | Spray-coating | 51%  81% | 625  8160 | 0.75  0.21 | [2] |
| Ti_3_C_2_T_x_ | ~80 nm | Spin-coating | 72%  83% | 2010  3850 | 0.53  0.50 | [3] |
| Ti_3_C_2_T_x_ | ~110 nm | Spin-coating | 77%  87% | 437  8900 | 0.29 | [4] |
| Ti_3_C_2_T_x_ | ~1 μm | Spin-coating | 65%  80%  86% | 128  507  1100 | 6.12  3.14  2.19 | [5] |
| Ti_3_C_2_T_x_ | ∼0.5 μm | Spin-coating | 87% | 424 | 6.2 | [6] |
| V_2_C_2_T_x_ | 0.5−1 μm | Spin-coating | 50% | 67 | 6.8 | [7] |
| Ti_3_C_2_T_x_ | N/A | Spin-coating | 86% | 330 | 7.3 | [8] |
| Ti_3_C_2_T_x_ | N/A | Vacuum-assisted filtration and tape-free transfer | 65% | 182.9 | 4.3 | [9] |
| Ti_3_C_2_T_x_ | 6 μm | Slot-die coating | 68%  87%  92.4% | 70  434  1083 | 11  (fitting) | [10] |
| Ti_3_C_2_T_x_ | 52.01 μm^2^ | Langmuir-Blodgett assembly | 82%  89% | 206  318 | 8.16  (fitting) | [11] |
| Ti_3_C_2_T_x_ | 4 μm | Scanning centrifugal casting | 45%  66%  82%  89%  93% | 34  78  163  403  1406 | 10.88  (fitting) | This work |

**Table S2.** Comparison of EMI shielding performance of transparent films

| **Functional filler** | **Substrate** | **Methods** | **Functional layer thickness, *t* (μm)** | **Transparency at 550 nm** | **EMI *SE***  **(dB)** | ***SE*/*t***  **(dB μm^−1^)** | **Frequency range (Hz)** | **References** |
| --- | --- | --- | --- | --- | --- | --- | --- | --- |
| ITO | PC | Magnetron sputtering | 0.103 | 70.8% | 19.2 | 186.4 | 8.2−12.4 G | [12] |
| ITO-Au-ITO | PC | Magnetron sputtering | 0.126 | 69.6% | 26.8 | 212.7 | 8.2−12.4 G | [12] |
| SWCNTs | PET | Spin-coating | 0.05 | 80% | 8 | 160 | 0.06−1.2 T | [13] |
| CNTs with Ni-Pd nanoparticles | Sapphire wafer | Spraying and plating | 0.1 | 71.4% | 21.4 | 214 | 8.2−12.4 G | [14] |
| CA/AgNWs | PU | Mayer-rod coating | / | 92% | 20.7 | / | 8.2−12.4 G | [15] |
| PES/AgNWs | PET | Drawn-down rod-coating | / | 85% | 15 | / | 8.2−12.4 G | [16] |
| PAM-LiCl | PDMS | In situ polymerization | 200 | 90% | 56.06 | 0.28 | 0.5−4.5 T | [17] |
| Wrinkled MXene | PDMS | Interfacial self-assembly | 0.008 | 72% | 5.6 | 700 | 0.2−10 T | [18] |
| Ti_3_C_2_T_x_ | Glass/silicon wafer/PET | Langmuir-Blodgett assembly | 0.0125 | 82% | 9.02 | 721.6 | 8.2−12.4 G | [11] |
| Ti_3_C_2_T_x_ | Glass/silicon wafer/PET | Langmuir-Blodgett assembly | 0.02 | 75% | 11.45 | 572.5 | 8.2−12.4 G | [11] |
| Ti_3_C_2_T_x_ | PET | Scanning centrifugal casting | 0.008 | 82% | 8.5  10.6  10.47 | 1062.5  1268  1252 | 0.03−3 G  8.2−12.4 G  0.5−3 T | This work |
| Ti_3_C_2_T_x_ | PET | Scanning centrifugal casting | 0.017 | 66% | 10.6  14.9  16.67 | 623.5  876  980 | 0.03−3 G  8.2−12.4 G  0.5−3 T | This work |

**SUPPLEMENTARY NOTES**

**Supplementary Note S1.** The thickness calculation of Ti_3_C_2_T_x_ MXene films.

According to the literature,[19] the thickness (*t*) of MXene films less than 150 nm can be calculated using Equation S1:

$t=-\frac{\ln T_{550}}{\alpha_{550}}$ (S1)

where *T*_550_ is the transmittance at 550 nm of the scanning centrifugal-coated Ti_3_C_2_T_x_ MXene films, and *α*_550_ is the absorption coefficient at the wavelength of 550 nm. For Ti_3_C_2_T_x_ MXene, the value of *α*_550_ is (2.4 ± 0.1) × 10^5^ cm^−1^.[5, 8]

**Supplementary Note S2.** Figure of merit (FoM).[20]

Since the *R_s_* is strongly dependent on the film transparency (*T*_550_), figure of merit (FoM) for transparent conductive films are widely used to evaluate their quality according to Equations S2 and S3:

$T_{550}=\left( 1+\frac{1}{2R_{s}}\sqrt{\frac{\mu_{0}}{\varepsilon_{0}}}\frac{\sigma_{op}}{\sigma_{DC}} \right)^{-2}=\left( 1+\frac{188.5}{R_{s}}\frac{\sigma_{op}}{\sigma_{DC}} \right)^{-2}$ (S2)

$\frac{1}{R_{s}}=FoM\frac{{T_{550}}^{-0.5}-1}{188.5}$ (S3)

where *μ*_0_ is the vacuum permeability (4π × 10^−7^ H m^−1^), *ε*_0_ is the vacuum permittivity (8.85 × 10^−12^ F m^−1^), *σ*_op_ is the optical conductivity, *σ*_DC_ is the direct current (DC) conductivity, and *σ*_DC_/*σ*_op_ is defined as the FOM value, where a higher FOM value indicates better film optoelectronic properties.

**Supplementary Note S3.** Calculation of electromagnetic shielding effectiveness (EMI SE).

The scattering parameters (*S*_11_ and *S*_21_) of each sample were recorded via a vector network analyzer to calculate the coefficients of reflection (*R*), absorption (*A*), and transmission (*T*) using the following Equations S4−S6:

$R=\left| S_{11} \right|^{2}$ (S4)

$T=\left| S_{21} \right|^{2}$ (S5)

$A=1-R-T$ (S6)

The total EMI shielding effectiveness (EMI *SE*_T_) can be written as Equation S7:

${SE}_{T}=10\log\left( \frac{1}{T} \right)=10log \left( \frac{1}{\left| S_{21} \right|^{2}} \right)$ (S7)

and the contributions from reflection (*SE*_R_) and absorption (*SE*_A_) can be expressed as Equations S8 and S9, respectively:

${SE}_{R}=10\log\left( \frac{1}{1-R} \right)=10log \left( \frac{1}{{1-\left| S_{11} \right|}^{2}} \right)$ (S8)

${SE}_{A}=10\log\left( \frac{1-R}{T} \right)=10log \left( \frac{1-\left| S_{11} \right|^{2}}{\left| S_{21} \right|^{2}} \right)$ (S9)

**SUPPLEMENTARY MOVIE**

**Movie S1.** The low-emissivity MXene/PET films emit less thermal radiation compared to high-emissivity substrates, enhancing contrast for clear pattern detection in dark environments.

**REFERENCES**

[1] D. Wen, X. Wang, L. Liu, C. Hu, C. Sun, Y. Wu, Y. Zhao, J. Zhang, X. Liu, G. Ying, Inkjet Printing Transparent and Conductive MXene (Ti_3_C_2_T_x_) Films: A Strategy for Flexible Energy Storage Devices, *ACS Appl. Mater. Interfaces* **2021**, *13* (15), 17766, https://doi.org/10.1021/acsami.1c00724.

[2] K. Hantanasirisakul, M. Q. Zhao, P. Urbankowski, J. Halim, B. Anasori, S. Kota, C. E. Ren, M. W. Barsoum, Y. Gogotsi, Fabrication of Ti_3_C_2_T_x_ MXene Transparent Thin Films with Tunable Optoelectronic Properties, *Adv. Electron. Mater.* **2016**, *2* (6), 1600050, https://doi.org/10.1002/aelm.201600050.

[3] M. Ebrahimi, C.-T. Mei, Optoelectronic Properties of Ti_3_C_2_T_x_ MXene Transparent Conductive Electrodes: Microwave Synthesis of Parent MAX Phase, *Ceram. Int.* **2020**, *46* (18), 28114, https://doi.org/10.1016/j.ceramint.2020.07.307.

[4] M. Mariano, O. Mashtalir, F. Q. Antonio, W.-H. Ryu, B. Deng, F. Xia, Y. Gogotsi, A. D. Taylor, Solution-Processed Titanium Carbide MXene Films Examined as Highly Transparent Conductors, *Nanoscale* **2016**, *8* (36), 16371, https://doi.org/10.1039/C6NR03682A.

[5] G. Ying, A. D. Dillon, A. T. Fafarman, M. W. Barsoum, Transparent, Conductive Solution Processed Spincast 2D Ti_2_CT_x_ (MXene) Films, *Mater. Res. Lett.* **2017**, *5* (6), 391, https://doi.org/10.1080/21663831.2017.1296043.

[6] S. Kumar, D. Kang, V. H. Nguyen, N. Nasir, H. Hong, M. Kim, D. C. Nguyen, Y.-j. Lee, N. Lee, Y. Seo, Application of Titanium-Carbide MXene-Based Transparent Conducting Electrodes in Flexible Smart Windows, *ACS Appl. Mater. Interfaces* **2021**, *13* (34), 40976, https://doi.org/10.1021/acsami.1c12100.

[7] G. Ying, S. Kota, A. D. Dillon, A. T. Fafarman, M. W. Barsoum, Conductive Transparent V_2_CT_x_ (MXene) Films, *FlatChem* **2018**, *8*, 25, https://doi.org/10.1016/j.flatc.2018.03.001.

[8] A. D. Dillon, M. J. Ghidiu, A. L. Krick, J. Griggs, S. J. May, Y. Gogotsi, M. W. Barsoum, A. T. Fafarman, Highly Conductive Optical Quality Solution-Processed Films of 2D Titanium Carbide, *Adv. Funct. Mater.* **2016**, *26* (23), 4162, https://doi.org/10.1002/adfm.201600357.

[9] M. Li, T. Cheng, G. Liu, H. Huang, K. Li, Y. Li, J. Yang, B. Huang, Infrared Anomalies in Ultrathin Ti_3_C_2_T_x_ MXene Films, *arXiv e-prints* **2023**, https://doi.org/10.48550/arXiv.2312.09573.

[10] T. Guo, D. Zhou, M. Gao, S. Deng, M. Jafarpour, J. Avaro, A. Neels, E. Hack, J. Wang, J. Heier, C. Zhang, Large-Area Smooth Conductive Films Enabled by Scalable Slot-Die Coating of Ti_3_C_2_T_x_ MXene Aqueous Inks, *Adv. Funct. Mater.* **2023**, *33* (15), 2213183, https://doi.org/10.1002/adfm.202213183.

[11] J. Xue, D. Liu, C. Li, Z. Zhu, Y. Sun, X. Gao, Q. Zheng, Multifunctional Transparent Conductive Films via Langmuir–Blodgett Assembly of Large MXene Flakes, *Mater. Horiz.* **2025**, *12*, 1155, https://doi.org/10.1039/d4mh01450b.

[12] N. Erdogan, F. Erden, A. T. Astarlioglu, M. Ozdemir, S. Ozbay, G. Aygun, L. Ozyuzer, ITO/Au/ITO Multilayer Thin Films on Transparent Polycarbonate with Enhanced EMI Shielding Properties, *Curr. Appl Phys.* **2020**, *20* (4), 489, https://doi.org/10.1016/j.cap.2020.01.012.

[13] M. A. Seo, J. H. Yim, Y. H. Ahn, F. Rotermund, D. S. Kim, S. Lee, H. Lim, Terahertz Electromagnetic Interference Shielding Using Single-Walled Carbon Nanotube Flexible Films, *Appl. Phys. Lett.* **2008**, *93* (23), 231905, https://doi.org/10.1063/1.3046126.

[14] J.-B. Park, H. Rho, A.-N. Cha, H. Bae, S. H. Lee, S.-W. Ryu, T. Jeong, J.-S. Ha, Transparent Carbon Nanotube Web Structures with Ni-Pd Nanoparticles for Electromagnetic Interference (EMI) Shielding of Advanced Display Devices, *Appl. Surf. Sci.* **2020**, *516*, 145745, https://doi.org/10.1016/j.apsusc.2020.145745.

[15] L.-C. Jia, D.-X. Yan, X. Liu, R. Ma, H.-Y. Wu, Z.-M. Li, Highly Efficient and Reliable Transparent Electromagnetic Interference Shielding Film, *ACS Appl. Mater. Interfaces* **2018**, *10* (14), 11941, https://doi.org/10.1021/acsami.8b00492.

[16] M. Hu, J. Gao, Y. Dong, K. Li, G. Shan, S. Yang, R. K.-Y. Li, Flexible Transparent PES/Silver Nanowires/PET Sandwich-Structured Film for High-Efficiency Electromagnetic Interference Shielding, *Langmuir* **2012**, *28* (18), 7101, https://doi.org/10.1021/la300720y.

[17] W. Xie, J. Xie, S. Li, J. Liu, X. Xiao, Q. Wen, T. Ding, Transparent and Durable Terahertz Absorber Based on Enhanced Wave-Ion Interaction, *Adv. Funct. Mater.* **2024**, 2418541, https://doi.org/10.1002/adfm.202418541.

[18] S. Yang, Z. Lin, X. Wang, J. Huang, R. Yang, Z. Chen, Y. Jia, Z. Zeng, Z. Cao, H. Zhu, Y. Hu, E. Li, H. Chen, T. Wang, S. Deng, X. Gui, Stretchable, Transparent, and Ultra-Broadband Terahertz Shielding Thin Films Based on Wrinkled MXene Architectures, *Nano-Micro Lett.* **2024**, *16* (1), 165, https://doi.org/10.1007/s40820-024-01365-w.

[19] a) M. Han, C. E. Shuck, R. Rakhmanov, D. Parchment, B. Anasori, C. M. Koo, G. Friedman, Y. Gogotsi, Beyond Ti_3_C_2_T_x_: MXenes for Electromagnetic Interference Shielding, *ACS Nano* **2020**, *14* (4), 5008, https://doi.org/10.1021/acsnano.0c01312; b) G. V. Rogozhkin, N. E. Gordeev, H. A. Butt, V. A. Kondrashov, A. E. Goldt, V. A. Dmitrieva, A. R. Vildanova, S. D. Konev, I. V. Sergeichev, Z. Wang, J. Qi, Y. Yan, D. V. Adamchuk, S. A. Maksimenko, D. V. Krasnikov, A. G. Nasibulin, Mechanically Neutral and Facile Monitoring of Thermoset Matrices with Ultrathin and Highly Porous Carbon Nanotube Films, *Carbon* **2024**, *230*, 119603, https://doi.org/10.1016/j.carbon.2024.119603.

[20] M. Guo, Y. Yang, Z. Qu, X. Zheng, J. Gao, F. Min, W. Lv, L. Guo, Y. Qiao, Y. Song, Permeable Film Enabled Evaporation Control of Liquid-Assisted Assembly for Printing High-Uniformity Functional Patterns, *Adv. Funct. Mater.* **2024**, *35* (10), 2416447, https://doi.org/10.1002/adfm.202416447.
